# High-Resolution Swin Transformer for Automatic Medical Image Segmentation

**DOI:** 10.3390/s23073420

**Published:** 2023-03-24

**Authors:** Chen Wei, Shenghan Ren, Kaitai Guo, Haihong Hu, Jimin Liang

**Affiliations:** 1 College of Economics and Management, Xi’an University of Posts & Telecommunications, Xi’an 710061, China; weichen@xupt.edu.cn; 2 School of Life Science and Technology, Xidian University, Xi’an 710071, China; 3 School of Electronic Engineering, Xidian University, Xi’an 710071, China

**Keywords:** Transformer, Swin Transformer, self-attention, medical image segmentation

## Abstract

The resolution of feature maps is a critical factor for accurate medical image segmentation. Most of the existing Transformer-based networks for medical image segmentation adopt a U-Net-like architecture, which contains an encoder that converts the high-resolution input image into low-resolution feature maps using a sequence of Transformer blocks and a decoder that gradually generates high-resolution representations from low-resolution feature maps. However, the procedure of recovering high-resolution representations from low-resolution representations may harm the spatial precision of the generated segmentation masks. Unlike previous studies, in this study, we utilized the high-resolution network (HRNet) design style by replacing the convolutional layers with Transformer blocks, continuously exchanging feature map information with different resolutions generated by the Transformer blocks. The proposed Transformer-based network is named the high-resolution Swin Transformer network (HRSTNet). Extensive experiments demonstrated that the HRSTNet can achieve performance comparable with that of the state-of-the-art Transformer-based U-Net-like architecture on the 2021 Brain Tumor Segmentation dataset, the Medical Segmentation Decathlon’s liver dataset, and the BTCV multi-organ segmentation dataset.

## 1. Introduction

Although convolutional neural networks (CNNs) have been widely applied to different computer vision (CV) tasks, CNN drawbacks still exist, such as the locality properties of convolutional kernels preventing CNNs from providing a richer representation of contextual information for pixels. The performance of CV tasks may be compromised by the induction bias of CNNs, especially for dense prediction tasks. Transformer, which has been successfully used in natural language processing [1,2], excels at establishing the long-range dependencies of different words in a sentence. The strengths of Transformer have attracted the attention of CV researchers. In recent years, a collection of studies has emerged that utilizes Transformer to improve the performance of image classification [3,4], object detection [5], semantic segmentation [6], representation learning [7], and medical image segmentation [8,9,10,11,12].

Because of the large number of medical images generated every day, there is an urgent need for automated medical image segmentation to help doctors diagnose diseases. Existing medical image segmentation models [8,9,10,11,12] usually adopt a U-Net-like architecture to generate semantically meaningful representations for segmentation by gathering the local and global information for each pixel in the medical image. The U-Net-like architecture consists of an encoder and a decoder. A medical image is passed through the encoder, which gradually generates spatially reduced and semantically richer low-resolution representations containing global contextual information, while the decoder takes the low-resolution representations as input and generates high-resolution representations for segmentation. To further improve the global contextual representation information, some recent works replaced the CNN layers in the U-Net-like architecture with Transformer, with the new Transformer-based architectures [9,10,12] achieving state-of-the-art performance on several medical image benchmarks.

Even though a U-Net-like architecture can provide rich contextual information, the procedure of recovering high-resolution representations from low-resolution representations may harm the spatial precision of the generated segmentation masks. The HRNet [13] maintains the high-resolution representations and connects the high- and low-resolution representations in parallel, which can remove the necessity to recover high-resolution representations from low-resolution representations and hence generate more precise spatial representations. Unlike existing Transformer-based methods for medical image segmentation, which all adopt U-Net-like architectures, in this paper, we propose a high-resolution Swin Transformer network (HRSTNet) that combines Transformer with the HRNet architecture for volumetric medical image segmentation. We employ the Swin Transformer block [14] to generate parallel feature representations, utilize patch-merging and expanding blocks to downsample and upsample feature representations, and design a multi-resolution feature fusion block to fuse features of different resolutions.

The main contributions of this paper are threefold:

(1) We propose a new network, the HRSTNet, that combines the HRNet with Swin Transformer blocks for medical image segmentation. The Swin Transformer blocks generate parallel features of different resolutions, and the high-resolution features are maintained throughout the network to provide more precise spatial information.
(2) A multi-resolution feature fusion block is designed to generate contextual information-augmented representations, utilizing a patch-merging block to downsample feature maps and a patch-expanding block to upsample feature maps.
(3) We conduct extensive experiments on the 2021 Brain Tumor Segmentation (BraTS 2021) dataset [15,16,17,18,19], Medical Segmentation Decathlon (MSD)’s liver dataset [20,21], and the BTCV multi-organ segmentation dataset [22,23,24,25,26,27,28] to demonstrate that the HRSTNet can achieve comparable or even better performance than the recently proposed medical image segmentation methods. Based on the outcomes of the experimental results, our manuscript proposed a new effective Transformer-based medical image segmentation architecture.

## 2. Related Work

### 2.1. Vision Transformers

Inspired by the success of Transformer in natural language processing tasks, ViT [3] splits the input image into 16×16 patches and converts the patches into tokens. Then, an additional learnable classification token, together with the image tokens, is passed through the Transformer encoder. The feature of the classification token is used to perform the classification task. Through pretraining on a large dataset containing 303 million high-resolution images and fine-tuning to a smaller dataset, ViT can achieve comparable performance with CNNs. DeiT [4] designs a distillation method and a collection of training strategies that enable ViT to achieve comparable performance with CNNs without the need for pretraining on large datasets. The Swin Transformer [14] divides the patches into non-overlapping windows and restricts the self-attention calculation in the small or shifted windows, which introduces locality inductive bias. It also utilizes patch-merging layers to generate hierarchical representations that can benefit the downstream tasks, such as object detection or semantic segmentation. HRFormer [29] and HRViT [30] borrow the network architecture from the HRNet [13] and design a Transformer-based architecture that maintains the high-resolution features and exchanges information from different resolutions. HRFormer utilizes local window self-attention to generate features of different resolutions. However, the features may lack contextual information from other windows. HRViT proposes an HRViTAttn block to generate different resolution features and a MixCFN block to exchange information from features of different resolutions. Unlike HRFormer and HRViT, which are designed for two-dimensional (2D) image segmentation tasks, we utilize the Swin Transformer block to create an HRNet-like architecture and apply it to volumetric medical image segmentation tasks.

### 2.2. Medical Image Segmentation

Prior to deep learning, the organ atlas, intensity properties, radial projection, or wavelet transform are utilized to implement medical image segmentation. Prastawa et al. [31] converted brain tumor segmentation to outlier detection and used brain atlases to detect abnormal areas. You et al. [32] adopted radial projection and a modified steerable complex wavelet to implement retinal blood vessel segmentation.

Due to the excellent performance of the U-Net architecture [33] for medical image segmentation, lots of the CNN-based [34,35] and almost all recently proposed Transformer-based medical image segmentation methods [8,9,10,12,36,37,38] adopted U-Net-like architectures to capture the global and local contextual information for each pixel. Futrega et al. [34] carried out extensive ablation studies on several tricks to improve the performance of the U-Net architecture. By combining the selected effective tricks, their optimized U-Net architecture took third place in the test phase of the BraTS 2021 [15,16,17,18,19] segmentation competition. Luu et al. [35] experimented with several modifications of the nn-UNet [39], and the modified nn-UNet won first place in the test phase of the BraTS 2021 segmentation competition. UNETR [8] uses ViT as the encoder and converts the features from four different stages of ViT to build hierarchical feature maps, which are fed into the decoder to generate the segmentation mask. The nnFormer [12] consists of a Transformer-based encoder, a Transformer-based decoder, and a bottleneck block. The encoder and decoder focus on extracting local information by using the local self-attention layers, while the bottleneck block is in charge of gathering the global contextual information. TransBTS [37], CoTr [36], and U-Transformer [38] use a transformer to enhance the global contextual information of the features generated by a CNN. Among them, TransBTS and U-Transformer utilize the standard transformer layer, while CoTr designs a DeTrans layer that pays attention to a small set of key positions to reduce the computational cost. VT-UNet [9] adopts the Swin Transformer block to generate hierarchical representation features and proposes a decoder block to merge the features from the encoder and decoder. Similar to UNETR, Swin UNETR [10] designs an architecture but utilizes the Swin Transformer as an encoder. Moreover, similar to VT-UNet and Swin UNETR, we adopt the Swin Transformer block to extract features. However, in contrast to the recently proposed Transformer-based medical image segmentation methods, we follow the HRNet architecture and maintain the high-resolution features to provide precise spatial information.

## 3. Methods Section

Inspired by the HRNet, the proposed HRSTNet maintains a high-resolution representation and continually exchanges information from different resolution features. We delve into the details of the more spatially precise and contextually informative HRSTNet in this section.

### 3.1. Architecture Overview

**Figure 1:**
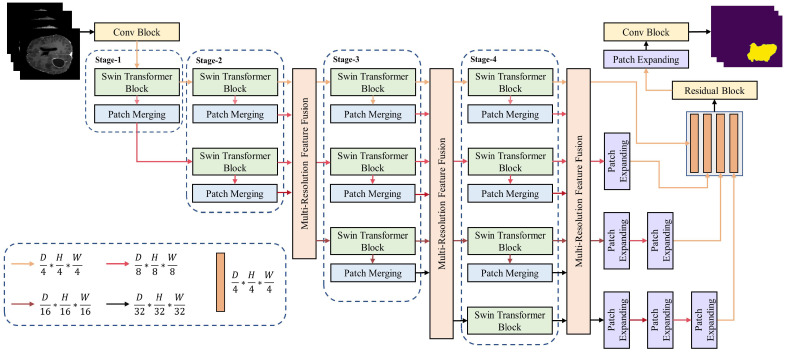
Overview of the HRSTNet.

The HRSTNet architecture, exemplified by a four-stage structure, is illustrated in Figure 1. It contains multiple sequentially connected stages with a multi-resolution feature fusion module inserted after each stage, except for the first stage. The *n*th stage contains *n* parallel Swin Transformer blocks that produce *n* parallel semantically richer low-resolution features. The features with the highest resolution are maintained in all the stages. By using a 3D convolutional block (Conv block), the input 3D image is split into small 3D patches, with each represented as a vector in the feature space. The feature vectors are passed from stage to stage, with the outputs of the last stage being aggregated by the last multi-resolution feature fusion block (MRFF). The outputs of the last feature fusion block are concatenated after the low-resolution features have been upsampled. The final segmentation mask is generated by passing the concatenated feature through a residual block, a patch-expanding block, and a Conv block.

The patch-merging block and patch-expanding block are used for down- and upsampling the feature maps, respectively. The definition of the patch-merging block is the same as the Swin Transformer [14], while the patch-expanding block is the same as VT-Unet [9]. The detailed structures of the Swin Transformer block, the HRSTNet stage, and the MRFF block are introduced in the following subsections.

### 3.2. Swin Transformer Block

**Figure 2:**
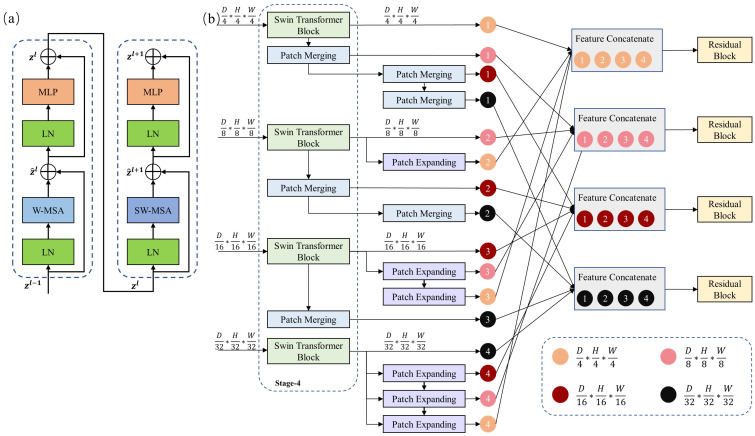
(**a**) The Swin Transformer block. (**b**) The multi-resolution feature fusion (MRFF) block.

The Swin Transformer block was introduced in previous studies [14,40], and it is illustrated in Figure 2a. It contains two cascading layers. The first layer calculates the self-attention in a small window (W-MSA) of the input 3D patches, and the second layer calculates the self-attention in the shifted window (SW-MSA) of the outputs of the previous layer.

Assume that the input 3D image is $X\in R^{D\times H\times W\times K}$, where D×H×W is the spatial size and *K* is the number of channels of the 3D data. The input *X* is fed into a Conv block with a kernel size P×P×P, stride *P*, and number of channels *C*. The Conv block splits *X* into small patches with a patch size of P×P×P. There are $S=\left\lfloor \frac{D}{P}\right\rfloor \times \left\lfloor \frac{H}{P}\right\rfloor \times \left\lfloor \frac{W}{P}\right\rfloor$ patches in total. The output of the Conv block can be denoted as $Z\in R^{\left\lfloor \frac{D}{P}\right\rfloor \times \left\lfloor \frac{H}{P}\right\rfloor \times \left\lfloor \frac{W}{P}\right\rfloor \times C}$. Because each pixel in *Z* corresponds to a patch-embedding vector, we resize the feature map *Z* into a 2D matrix $\vec{Z}\in R^{S\times C}$, in which each row represents a patch-embedding vector. For simplicity, we still use *Z* to represent $\vec{Z}$ in the following.

After patch embedding, the set of patch-embedding vectors is fed into the Swin Transformer block of the first stage (compare with Figure 1). The output of the Swin Transformer block is calculated as follows (compare with Figure 2a):

(1) $$\begin{array}{cc} \; & {\hat{Z}}^{l}=\mathrm{W-MSA}\left(\mathrm{LN}\left(Z^{l-1}\right)\right)+Z^{l-1}, \end{array}$$

(2) $$\begin{array}{cc} \; & Z^{l}=\mathrm{MLP}\left(\mathrm{LN}\left({\hat{Z}}^{l}\right)\right)+{\hat{Z}}^{l}, \end{array}$$

(3) $$\begin{array}{cc} \; & {\hat{Z}}^{l+1}=\mathrm{SW-MSA}\left(\mathrm{LN}\left(Z^{l}\right)\right)+Z^{l}, \end{array}$$

(4) $$\begin{array}{cc} \; & Z^{l+1}=\mathrm{MLP}\left(\mathrm{LN}\left({\hat{Z}}^{l+1}\right)\right)+{\hat{Z}}^{l+1}, \end{array}$$

where the definitions of W-MSA, SW-MSA, LN, and MLP are the same as in the Swin Transformer [14]. The Swin Transformer blocks of other stages work similarly, except that their input is the output of the former stage.

### 3.3. HRSTNet Stage

The HRSTNet contains multiple stages that generate multi-resolution feature maps. Except for the last stage, the *n*-th stage contains *n* Swin Transformer blocks and *n* patch-merging blocks, while the last stage contains n−1 patch-merging blocks. In the *n*-th stage, the *n* parallel Swin Transformer blocks process *n* different resolution feature maps, respectively. Each Swin Transformer block is followed by a patch merge, which is used to downsample the output feature map of the Swin Transformer block.

Taking the third stage (Stage 3, compare with Figure 1) as an example, three feature maps with a resolution of $\frac{D}{4}\times \frac{H}{4}\times \frac{W}{4}$, $\frac{D}{8}\times \frac{H}{8}\times \frac{W}{8}$, and $\frac{D}{16}\times \frac{H}{16}\times \frac{W}{16}$ are input into this stage. Three Swin Transformer blocks process the input feature maps independently and output feature maps with the same resolution as their input. The outputs of the Swin Transformers are further downsampled by the patch-merging layers separately, resulting in three feature maps with resolutions of $\frac{D}{8}\times \frac{H}{8}\times \frac{W}{8}$, $\frac{D}{16}\times \frac{H}{16}\times \frac{W}{16}$, and $\frac{D}{32}\times \frac{H}{32}\times \frac{W}{32}$. The feature map with a resolution of $\frac{D}{32}\times \frac{H}{32}\times \frac{W}{32}$ is directly fed into the fourth stage (Stage 4), while the outputs of other patch-merging layers and the outputs of the Swin Transformer blocks are fed into an MRFF block.

### 3.4. Multi-Resolution Feature Fusion

The multi-resolution feature fusion block is used to exchange information from feature maps with different resolutions and produce new feature maps that are spatially more precise and semantically richer. Figure 2b shows the MRFF block following the fourth stage, which aggregates the features of four different resolutions.

The fourth stage of the HRSTNet contains four Swin Transformer blocks, which generate four feature maps with resolutions of $\frac{D}{4}\times \frac{H}{4}\times \frac{W}{4}$, $\frac{D}{8}\times \frac{H}{8}\times \frac{W}{8}$, $\frac{D}{16}\times \frac{H}{16}\times \frac{W}{16}$, and $\frac{D}{32}\times \frac{H}{32}\times \frac{W}{32}$. The feature maps are referred to as $f_{4},f_{8},f_{16}$, and $f_{32}$, respectively. Each of the four feature maps is down- or upsampled to the other three resolutions. Taking $f_{4}$ as an example, it sequentially passes through three patch-merging layers to generate three new feature maps with resolutions of $\frac{D}{8}\times \frac{H}{8}\times \frac{W}{8}$, $\frac{D}{16}\times \frac{H}{16}\times \frac{W}{16}$, and $\frac{D}{32}\times \frac{H}{32}\times \frac{W}{32}$. The resultant feature maps are denoted as $f_{4\_8}$, $f_{4\_16}$, and $f_{4\_32}$, respectively. The feature maps generated by $f_{8}$, $f_{16}$, and $f_{32}$ can be easily inferred and are denoted as $\left\{f_{8\_4},f_{8\_16},f_{8\_32}\right\}$, $\left\{f_{16\_4},f_{16\_8},f_{16\_32}\right\}$, and $\left\{f_{32\_4},f_{32\_8},f_{32\_16}\right\}$, respectively. We then concatenate the feature maps with the same resolution (e.g., $\left[f_{4},f_{8\_4},f_{16\_4},f_{32\_4}\right]$, where the symbol [] indicates feature concatenation). The concatenated feature maps are fed into a residual block to produce the output of the MRFF block, resulting in four feature maps with resolutions of $\frac{D}{4}\times \frac{H}{4}\times \frac{W}{4}$, $\frac{D}{8}\times \frac{H}{8}\times \frac{W}{8}$, $\frac{D}{16}\times \frac{H}{16}\times \frac{W}{16}$, and $\frac{D}{32}\times \frac{H}{32}\times \frac{W}{32}$.

The outputs of the MRFF blocks after the second and third stages are derived in a similar manner to that described above. The outputs of the last MRFF block with the resolution lower than $\frac{D}{4}\times \frac{H}{4}\times \frac{W}{4}$ are upsampled (compare with Figure 1) and concatenated to generate the output segmentation mask.

Except for using the last MRFF block (shown in Figure 1), we can also use the outputs of the MRFF block after the second or the third stage to generate the segmentation mask. The corresponding architectures are denoted as HRSTNet-2 and HRSTNet-3, respectively. The architecture shown in Figure 1 is denoted as HRSTNet-4.

## 4. Experiments and Results

In this section, we evaluate the performance of the HRSTNet on the BraTS 2021 dataset [15,16,17,18,19], the MSD liver dataset [20,21], and the BTCV multi-organ segmentation challenge [22,23,24,25,26,27,28]. All the experiments were implemented with Pytorch [41] on two NVIDIA GeForce RTX 3090 graphic cards. We utilized MONAI (https://monai.io/, accessed on 16 April 2022), a healthcare imaging processing framework, for network training and data preprocessing. The 3D Slicer [42,43] software was employed to visualize the experimental results. The code of HRSTNet is publicly available at https://github.com/auroua/HRSTNet.

### 4.1. Implementation Details

The BraTS 2021 dataset contains 1251 magnetic resonance imaging (MRI) scans in the shape of 240×240×155. Following the VT-UNet [9], the 1251 scans were split into groups of 834, 208, and 209 for training, validation, and testing, respectively. The task was to segment three semantically meaningful tumor classes, namely the enhanced tumor (ET), tumor core (TC) region, and whole tumor (WT) region. The MSD liver dataset contains 131 computed tomography (CT) volumes, which were split into groups of 87, 22, and 22 for training, validation, and testing, respectively. The task for the liver dataset was tumor segmentation. The BTCV multi-organ segmentation dataset [22,23,24,25,26,27,28] contains 50 clinical CT volumes, and the task of this dataset was to automatically segment 13 abdominal organs (adrenal, aorta, esophagus, gallbladder, kidney, liver, pancreas, spleen and portal vein, spleen, stomach, and vena cava). The 50 CT volumes were split into 2 parts for training and testing, and the 2 parts contained 30 and 20 CT volumes, respectively. We selected 24 CT volumes from the training dataset to train the segmentation algorithm, and the remaining 6 CT volumes were used to evaluate the performance of the algorithms.

The depth of the Swin Transformer blocks in all the stages was 2, and the heads of the Swin Transformer blocks for resolutions of $\frac{D}{4}\times \frac{H}{4}\times \frac{W}{4}$, $\frac{D}{8}\times \frac{H}{8}\times \frac{W}{8}$, $\frac{D}{16}\times \frac{H}{16}\times \frac{W}{16}$, and $\frac{D}{32}\times \frac{H}{32}\times \frac{W}{32}$ were 3, 6, 12, and 24, respectively. The AdamW optimizer was used to optimize the parameters of the HRSTNet, and the initial learning rate was $1\times 10^{-4}$. The cosine decay learning rate scheduler [44] with a linear warm-up was used to adjust the value of the learning rate, and the warm-up epoch was 50. The sum of the dice loss with the cross-entropy loss was adopted as the loss function.

The MRI data from BraTS 2021 were cropped to 128×128×128. Three Transformer-based architectures (UNETR [8], Swin UNETR [10], and VT-UNet-B [9]) and two CNN-based architectures (optimized UNet [34] and extending nnUNet [35]) were compared on this dataset.

The CT volumes from the liver dataset were cropped to 96×96×96. The other training details were the same as those of the BraTS 2021 dataset. Because the splitting method for the liver dataset used in this experiment was the same as the one for VT-UNet, we compared the HRSTNet with the methods listed in the VT-Net, including 3D UNet [11], nnFormer [12], and VT-UNet [9]. We obtained their experiment results from [9].

The CT volumes from the BTCV multi-organ segmentation dataset were also cropped to 96×96×96, and the HRSTNet was compared with UNETR, VT-UNet, and Swin UNETR. We retrained all the models for 1000 epochs on this dataset, and the batch size was set to 1. The learning rate of the HRSTNet trained on the BTCV dataset was set to $1\times 10^{-3}$.

### 4.2. Evaluation Metrics

The Dice similarity coefficient (DCS) and 95% Hausdorff distance (HD95) are used to evaluate the performance of different algorithms on the BraTS 2021 dataset, while the DCS is used to compare algorithms on the MSD liver dataset and BTCV multi-organ segmentation dataset.

The DCS is a commonly used evaluation metric in medical image segmentation which measures the overlap between the predicted segmentation mask and the ground truth segmentation mask. It can be formulated as follows:

(5) $$DCS\left(P,G\right)=\frac{2\times \left|P\cap G\right|}{\left|P\right|+\left|G\right|}$$

where *P* is the predicted binary segmentation mask, *G* is the ground truth binary segmentation mask, and the symbol | | is used to sum the nonzero region.

HD95 is always utilized to measure the distance between the boundary of the predicted binary segmentation mask and the boundary of the ground truth binary segmentation mask. The Hausdorff distance (HD) can be formulated as follows:

(6) $$H\left(P,G\right)=\max\{\max_{p_{b}\in P}\left\{\min_{g_{b}\in G}∥p_{b}-g_{b}∥\right\},\max_{g_{b}\in G}\left\{\min_{p_{b}\in P}∥p_{b}-g_{b}∥\right\}\}$$

where $p_{b}$ is a pixel in the boundary of the predicted segmentation mask and $g_{b}$ is a pixel in the boundary of the ground truth segmentation mask. The HD reflects the distance between the boundaries of the two segmentation masks. To reduce the effect of outlier pixels, the 95th percentile of the Hausdorff distance (HD) is used in medical image segmentation to measure the distance between boundaries, a value known as HD95.

During training, the model with the best Dice similarity coefficient (DSC) on the validation dataset was saved for the final evaluation, and model evaluation tricks, such as model ensembles, were not used in this paper.

### 4.3. Ablation Study of the MRFF Block

Because the MRFF block utilizes several feature maps of different resolutions to generate the final segmentation mask, experiments were conducted to determine which combination of feature maps resulted in the best HRSTNet performance. We compared the MRFF block when utilizing a single feature map ($\frac{D}{16}\times \frac{H}{16}\times \frac{W}{16}$), two feature maps ($\frac{D}{16}\times \frac{H}{16}\times \frac{W}{16}$ and $\frac{D}{32}\times \frac{H}{32}\times \frac{W}{32}$), three feature maps ($\frac{D}{8}\times \frac{H}{8}\times \frac{W}{8}$, $\frac{D}{16}\times \frac{H}{16}\times \frac{W}{16}$, and $\frac{D}{32}\times \frac{H}{32}\times \frac{W}{32}$), and four feature maps ($\frac{D}{4}\times \frac{H}{4}\times \frac{W}{4}$, $\frac{D}{8}\times \frac{H}{8}\times \frac{W}{8}$, $\frac{D}{16}\times \frac{H}{16}\times \frac{W}{16}$, and $\frac{D}{32}\times \frac{H}{32}\times \frac{W}{32}$), and the experimental results are illustrated in Table 1. The experimental results show that the performance of HRSTNet-4 improved as the MRFF block combined more feature maps. The experimental results show that utilizing more feature maps with different resolutions could provide diverse information and hence enhance the final performance. Because HRSTNet-4 with the MRFF block utilizing four different feature maps achieved the best performance, the experiment conducted in the following subsection uses an MRFF block with four different resolutions.

### 4.4. Experimental Results on BraTS 2021

The DCS and HD95 were used to quantitatively evaluate the segmentation results on the BraTS 2021 dataset. We utilized the method (https://github.com/MIC-DKFZ/nnUNet/blob/5c18fa32f2b31575aae59d889d196e4c4ba8b844/nnunet/dataset_conversion/Task082_BraTS_2020.py#L330, accessed on 1 July 2022) provided by the nnU-Net [39] to calculate the Hausdorff distance. The FLOPs were calculated by using MMCV [45], and the size of the input tensor was set to (4, 128, 128, 128) when calculating the FLOPs.

The experimental results on the BraTS 2021 dataset are presented in Table 2. Since the CNN-based UNet has been thoroughly studied for several years, and many tricks have been proposed to improve the performance of CNN-based UNet architectures, the CNN-based extending nnUNet achieved the highest performance. The extending nnUNet had the highest FLOP count, which means the inference speed of this model may be slower than the other compared models.

Among the Transformer-based architectures, our proposed HRSTNet-4 achieved the best average Dice score, while HRSTNet-3 obtained the smallest average Hausdorff distance. Even the HRSTNet-2 method, which had the lowest FLOP count among the series of HRSTNets, had a better average Hausdorff distance compared with the other three Transformer-based methods, and its average Dice score was comparable to that of VT-UNet. For enhancing tumor segmentation, VT-UNet-B performed best in terms of Dice score among the Transformer-based architectures, but its Hausdorff distance was the largest among all the other Transformer-based models. Swin UNETR had a comparable Dice score with UNETR, but its Hausdorff score is better than that of UNETR. Considering that the difference between these two networks is the encoder, we conjecture that it was the Transformer’s ability to construct long-term dependencies that led to the improvement. Overall, the experimental results illustrated in Table 2 indicate that among the Transformer-based architectures, the HRNet-like network can achieve better performance than the U-Net-like architectures, which implies that HRNet-like network design is a valuable future research direction.

We emphasize that this study aims to provide a new Transformer-based medical image segmentation architecture which is different from existing U-Net-like Transformer-based architectures and to verify the effectiveness of the designed architecture. Extensive ablation studies on different tricks to enhance the performance of the HRSTNet are left for further investigation.

**Figure 3:**
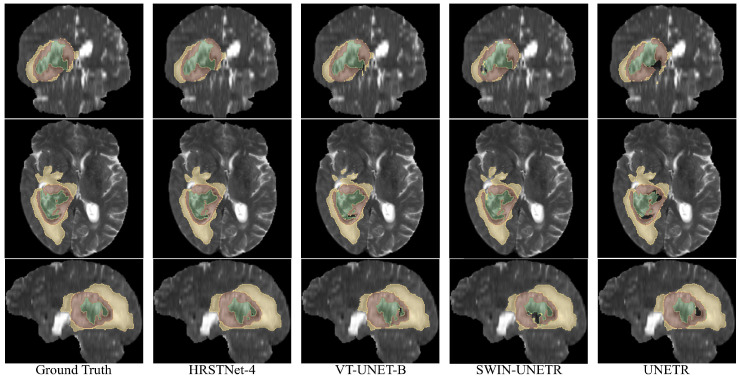
Visual examples of the segmentation results on BraTS 2021. The top through bottom rows correspond to the coronal, axial, and sagittal views, respectively. The green, brown, and yellow colors represent the necrotic tumor core (NCR), enhancing tumor (ET), and peritumoral edematous (ED), respectively.

Figure 3 shows the visualization of the segmentation results on BraTS 2021. As shown in the last column of Figure 3, UNETR performed well when segmenting the ET (brown) and ED (yellow), but when segmenting the NCR (green), as shown by the black hole inside the brown area, UNETR failed to segment NCR correctly. From the sagittal view of the Swin-UNETR segmentation results, we can find a large black hole within the brown area, which means that Swin-UNETR failed to successfully segment all the pixels belonging to the NCR. As shown in the axial view of the VT_UNET-B segmentation results, the upper part of the segmented ED (yellow) region was significantly smaller compared with the ground truth. From the axial and sagittal views of the segmentation results of VT_UNET-B, we can also find that the segmentation mask of the NCR failed to include all the parts of ground truth NCR area. In summary, from Figure 3, we can visually find that the segmentation results of HRSTNet-4 were better than those of the other Transformer-based U-Net-like architectures.

### 4.5. Experimental Results on the MSD Liver Dataset

The Dice score was used to evaluate the segmentation results on the MSD liver dataset for liver tumor segmentation. Table 3 shows that the HRSTNet architectures (HRSTNet-2, HRSTNet-3, and HRSTNet-4) performed better than other algorithms on tumor segmentation, and HRSTNet-3 surpassed the existing methods by a large margin of +9.54 in tumor Dice score. In the series of HRSTNet architectures, HRSTNet-3 performed better than HRSTNet-2 and HRSTNet-4, and HRSTNet-4 performed better than HRSTNet-2. Compared with BraTS 2021, the MSD liver dataset is relatively small, and we argue that HRSTNet-4 suffered from overfitting, thus resulting in slightly worse performance. The results show that, except for MRI segmentation, the HRSTNet was also excellent in CT volume segmentation. Again, we would like to clarify that no model ensemble was used in the experiments conducted in this section.

Because the segmentation of liver tumors was much better than in the comparative methods, and the tumors were smaller compared with the organs, this suggests that maintaining a high-resolution feature map helps to improve the performance of small-target segmentation. We also conjecture that the continuous exchange of information from feature maps of different resolutions can improve the contextual information of feature maps, which is also critical for enhancing the segmentation performance of small targets. Further studies of the mechanisms of high-resolution feature map maintenance and the continuous exchange of information from feature maps with different resolutions is needed to provide guidelines for the design of a HRNet-like Transformer-based architecture for medical image segmentation.

For the organ segmentation, HRSTNet-2, HRSTNet-3, and HRSTNet-4 all performed worse than the other compared algorithms. We presume that this was caused by the inconsistent fusing of feature maps with different resolutions, and this issue is left open for further study.

**Figure 4:**
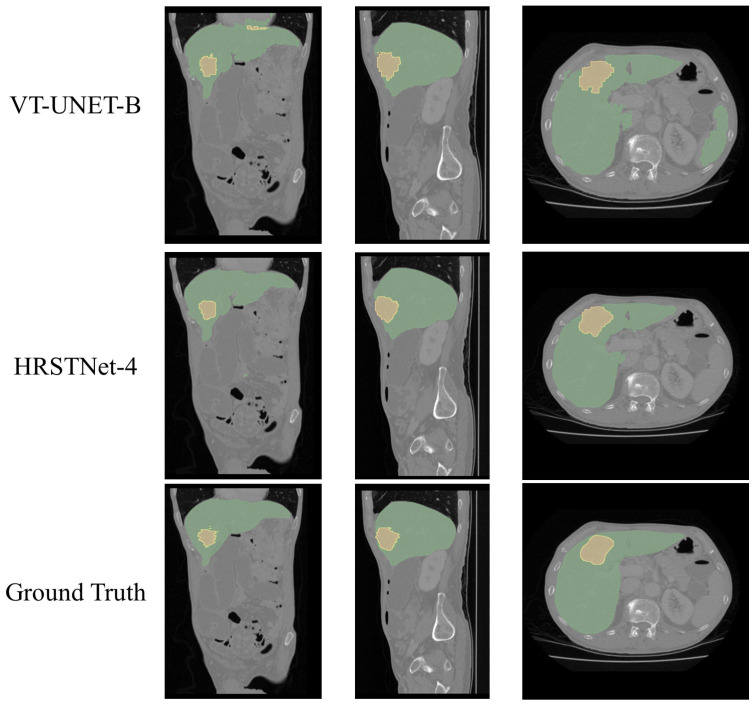
Visual examples of the segmentation results on the MSD liver dataset. The columns from left to right correspond to the coronal, sagittal, and axial views, respectively. The green and yellow colors represent the liver and the tumor, respectively.

The visualization of the segmentation results of HRSTNet-4 and VT-Unet on the MSD liver dataset is shown in Figure 4, which shows that HRSTNet-4 can segment the liver and tumor more accurately.

### 4.6. Experimental Results on the BTCV Multi-Organ Segmentation Dataset

The performance of the HRSTNet compared with other Transformer-based architectures on the BTCV multi-organ segmentation validation dataset is shown in Table 4. As with the MSD liver dataset, the Dice score was used to compare the performance of different algorithms. HRSTNet-4 performed better than UNETR and slightly worse than Swin UNETR and VT-UNet-B, and the performances of Swin UNETR and VT-UNet-B were nearly identical. Because the architectures’ validation performances were compared, and Swin UNETR had the largest quantity of FLOPs, Swin UNETR achieved the best average DICE score. HRSTNet-4 achieved the best performance for stomach and pancreas segmentation, while VT-UNet-B achieved the best performance in gallbladder and liver segmentation.

### 4.7. Model Efficiency Analysis

This subsection discusses the efficiency of the HRSTNet in terms of the training convergence rate, training time, and FLOPs. The data used to analyze the above aspects were collected from experiments conducted on the BraTS 2021 dataset.

**Figure 5:**
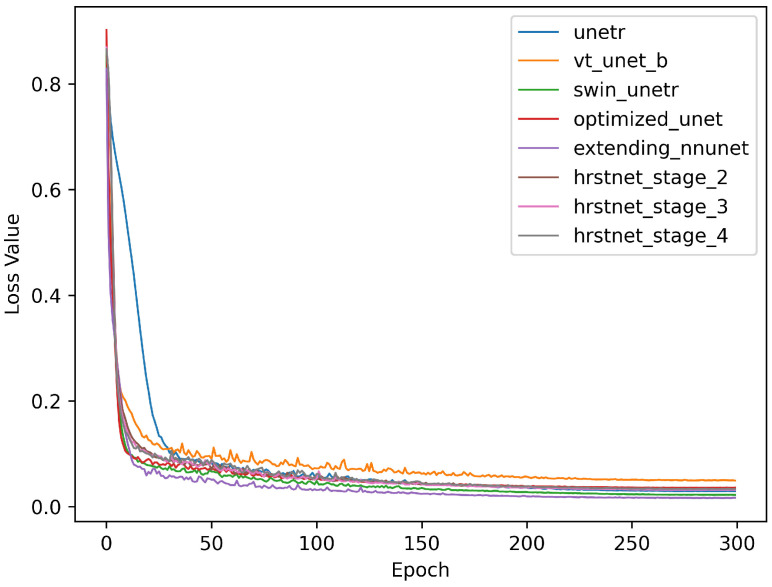
Comparison of training loss curves.

**Figure 6:**
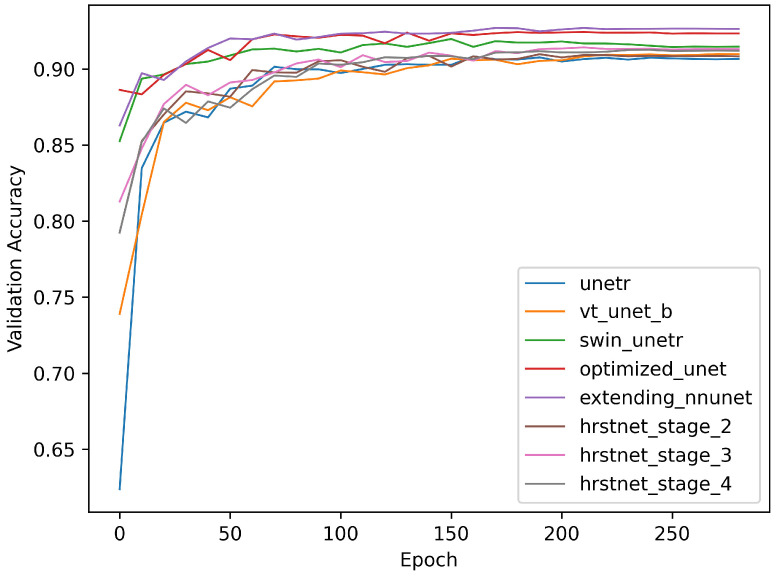
Comparison of validation accuracy curves.

Figure 5 and Figure 6 demonstrate the training loss and validation accuracy curves, respectively. Figure 5 shows that the convergence rate of the HRSTNet and other Transformer-based architectures was approximately the same as those of the CNN-based architectures, except for the slower convergence rate of UNETR. Figure 6 shows that all the models compared shared the same trend (i.e., the validation accuracy of the models progressively improved as the loss in the training process gradually decreased).

As shown in Table 2, the model size of HRSTNet-4 was larger than that of the CNN-based architecture, but the computational cost of HRSTNet-4 was lower than that of the CNN-based architecture. The comparison of the training time of one epoch for different models is shown in Table 5, and it shows that training HRSTNet-4 was more efficient than training the two CNN-based architectures.

The above analysis verifies that the HRSTNet had the same training efficiency as the CNN-based architecture. Since the FLOPs of the HRSTNet were lower than for the CNN-based architecture, the HRSTNet may have a faster inference speed than the CNN-based architecture.

## 5. Conclusions

In conclusion, we proposed an HRNet-like architecture, the HRSTNet, for medical image segmentation and validated its superior performance through experiments. We conjecture that maintaining the high-resolution feature map and continuously exchanging information from different feature maps of different resolutions can improve its performance. Maintaining a high-resolution feature map can provide more precise spatial details for generating segmentation masks, while the continuous exchange of information from different feature maps can provide more contextual information. The experimental results for the BraTS 2021 dataset show that the HRSTNet surpassed the existing Transformer-based methods by a margin of +1.8 for the average HD95 score, indicating that HRSTNet-generated masks were more tightly aligned with the ground truth. The experimental results for the MSD liver dataset illustrate that the HRSTNet achieved far better results than the existing methods for tumor segmentation. The experimental results for the BTCV multi-organ segmentation dataset demonstrate that the HRSTNet achieved a performance comparable to those of existing Transformer-based architectures.

Despite the benefits of the HRSTNet, more experiments are necessary to thoroughly verify its performance. To verify the generality, we will conduct experiments on more diverse datasets. To enhance the performance of the HRSTNet, pretraining strategies, such as self-supervised and masked image modeling, can be utilized to allow the model to learn meaningful knowledge for the downstream tasks. Tricks such as deep supervision loss, focal loss, decoder attention, and the combination of different kinds of attention strategies can be investigated to improve the performance of the HRSTNet further. Because this study has shown that the HRNet-like architecture is applicable to medical image segmentation, and there are many different HRNet-like architectures that can be designed, to effectively design Transformer-based HRNet-like architectures, we can design a search space and utilize the neural architecture search strategies to find the HRNet-like architecture with optimal performance.

## Figures and Tables

**Table 1:** Performance comparison of HRSTNet-4 using different feature maps on the BraTS 2021 dataset. The numbers listed in the symbol [] represent the feature maps used in the MRFF block.

Methods	Average		WT		ET		TC	
	HD95 ↓	DSC ↑	HD95 ↓	DSC ↑	HD95 ↓	DSC ↑	HD95 ↓	DSC ↑
HRSTNet-4-[16]	10.42	86.26	4.32	91.02	15.92	81.20	11.02	86.56
HRSTNet-4-[16, 32]	9.96	86.44	4.90	91.00	15.79	81.63	9.18	86.69
HRSTNet-4-[8, 16, 32]	9.07	87.10	4.34	91.72	**15.44**	82.29	7.41	87.29
HRSTNet-4-[4, 8, 16, 32]	**8.94**	**87.48**	**4.09**	**91.90**	15.62	**82.92**	**7.11**	**87.62**

**Table 2:** Performance comparison on BraTS 2021 dataset.

Methods	FLOPs(G)	Params(M)	Average		WT		ET		TC	
			HD95 ↓	DSC ↑	HD95 ↓	DSC ↑	HD95 ↓	DSC ↑	HD95 ↓	DSC ↑
Optimized UNet [34]	1345.57	51.23	10.62	87.84	5.66	92.21	17.18	83.67	9.04	87.65
Extending nnUNet [35]	1657.97	87.41	**2.99**	**92.69**	**3.62**	**93.40**	**2.50**	**90.79**	**2.86**	**93.89**
UNETR [8]	175.85	102.45	11.04	86.18	6.18	91.40	18.31	81.80	8.63	85.33
Swin UNETR [10]	1218.79 ^🟉^	240.96	9.84	86.13	6.06	90.42	15.27	83.36	8.19	84.62
VT-UNet-B [9]	124.03	20.76	13.36	87.31	8.96	91.20	18.69	83.85	12.44	86.87
HRSTNet-2	107.47	7.19	8.99	87.22	4.15	92.06	15.63	82.64	7.20	86.95
HRSTNet-3	202.76	50.07	8.06	86.94	4.79	91.81	13.91	82.20	5.50	86.80
HRSTNet-4	318.43	266.33	8.94	87.48	4.09	91.90	15.62	82.92	7.11	87.62

^🟉^ This value is obtained by setting the input size to 4 × 96 × 96 × 96.

**Table 3:** Performance comparison on the liver dataset.

Methods	Liver		
	Organ	Tumor	AVG.
3D UNet [11]	92.67	34.92	63.80
nnFormer [12]	89.43	31.84	60.63
VT-UNet-B [9]	**92.84**	35.69	**64.26**
HRSTNet-2	79.40	40.75	60.07
HRSTNet-3	82.40	**45.23**	63.81
HRSTNet-4	80.81	41.36	61.09

**Table 4:** Performance comparison of Dice scores on BTCV multi-organ segmentation dataset.

Methods	Spl	RKid	LKid	Gall	Eso	Liv	Sto	Aor	IVC	Veins	Pan	AG	Avg.
UNETR	0.926	0.888	0.860	0.390	0.713	0.953	0.687	0.897	0.802	0.703	0.621	0.556	0.735
Swin UNETR	**0.959**	**0.949**	**0.941**	0.442	**0.754**	0.963	0.707	**0.912**	**0.823**	**0.758**	0.692	**0.662**	**0.787**
VT-UNet-B	0.953	0.942	0.940	**0.568**	0.751	**0.964**	0.702	0.896	0.802	0.731	0.697	0.626	0.784
HRSTNet-4	0.945	0.934	0.936	0.457	0.689	0.962	**0.754**	0.881	0.785	0.718	**0.710**	0.610	0.769

Note: Spl = spleen, RKid = right kidney, LKid = left kidney, Gall = gallbladder, Eso = esophagus, Liv = liver, Sto = stomach, Aor = aorta, IVC = inferior vena cava, Veins = portal and splenic veins, Pan = pancreas, and AG =left and right adrenal glands.

**Table 5:** Comparison of training time of one epoch for different models.

Optimized UNet (S)	Extending nnUNet (S)	HRSTNet-4 (S)
412.82	443.85	389.45

S is the abbreviation for seconds.

## Data Availability

Three publicly available datasets were used in this manuscript: BraTS 2021, the MSD liver dataset, and the BTCV multi-organ segmentation dataset. These datasets can be found at https://www.med.upenn.edu/cbica/brats2021/, http://medicaldecathlon.com/, and https://zenodo.org/record/1169361#.Y-cPV3ZBy38 (accessed on 26 May 2022), respectively.

## Abbreviations

The following abbreviations are used in this manuscript:

HRNet	High-resolution network
HRSTNet	High-resolution Swin Transformer network
CNNs	Convolutional neural networks
CV	Computer vision
MSD	Medical Segmentation Decathlon
MRFF	Multi-resolution feature fusion block
SW-MSA	Self-attention in the shifted window
MRI	Magnetic resonance imaging
CT	Computed tomography
ET	Enhanced tumor
TC	Tumor core
WT	Whole tumor
DSC	Dice similarity coefficient
HD	Hausdorff distance
